# Genomic analyses reveal recurrent mutations in epigenetic modifiers and the JAK–STAT pathway in Sézary syndrome

**DOI:** 10.1038/ncomms9470

**Published:** 2015-09-29

**Authors:** Mark J. Kiel, Anagh A. Sahasrabuddhe, Delphine C. M. Rolland, Thirunavukkarasu Velusamy, Fuzon Chung, Matthew Schaller, Nathanael G. Bailey, Bryan L. Betz, Roberto N. Miranda, Pierluigi Porcu, John C. Byrd, L. Jeffrey Medeiros, Steven L. Kunkel, David W. Bahler, Megan S. Lim, Kojo S. J. Elenitoba-Johnson

**Affiliations:** 1 Department of Pathology, University of Michigan Medical School, Ann Arbor, Michigan 48109, USA; 2 Department of Pathology and Laboratory Medicine, Perelman School of Medicine at University of Pennsylvania, Philadelphia, Pennsylvania 19104, USA; 3 Department of Hematopathology, The University of Texas MD Anderson Cancer Center, Houston, Texas 77030, USA; 4 Division of Hematology, Department of Internal Medicine, The Ohio State University, Columbus, Ohio 43210, USA; 5 Department of Pathology, The University of Utah Health Sciences Center, Salt Lake City, Utah 84112, USA; 6 Center for Personalized Diagnostics, Perelman School of Medicine at University of Pennsylvania., Philadelphia, Pennsylvania 19104, USA

## Abstract

Sézary syndrome (SS) is an aggressive leukaemia of mature T cells with poor prognosis and limited options for targeted therapies. The comprehensive genetic alterations underlying the pathogenesis of SS are unknown. Here we integrate whole-genome sequencing (*n*=6), whole-exome sequencing (*n*=66) and array comparative genomic hybridization-based copy-number analysis (*n*=80) of primary SS samples. We identify previously unknown recurrent loss-of-function aberrations targeting members of the chromatin remodelling/histone modification and trithorax families, including *ARID1A* in which functional loss from nonsense and frameshift mutations and/or targeted deletions is observed in 40.3% of SS genomes. We also identify recurrent gain-of-function mutations targeting *PLCG1* (9%) and *JAK1*, *JAK3*, *STAT3* and *STAT5B* (*JAK/STAT* total ∼11%). Functional studies reveal sensitivity of JAK1-mutated primary SS cells to JAK inhibitor treatment. These results highlight the complex genomic landscape of SS and a role for inhibition of JAK/STAT pathways for the treatment of SS.

## Abstract (web summary)

Sézary syndrome is a T cell malignancy that has been poorly characterized at the genome level. In this study, Kiel *et al*. perform whole-genome analyses and identify mutations in the JAK–STAT pathway and show that primary cells are sensitive to JAK inhibitors.

## Graphical abstract

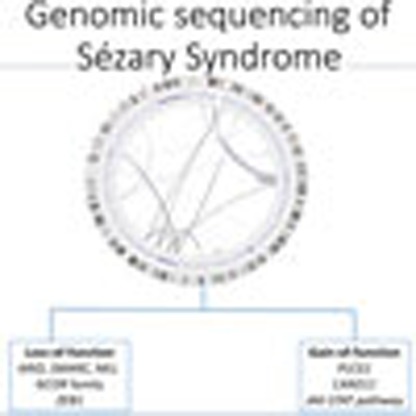

Sézary syndrome (SS) is an aggressive mature T-cell leukaemia with a median 5-year survival rate of <20% (refs 1, 2). The skin is almost always affected, whereas in advanced forms of SS lymph nodes and other visceral organs can be involved^3^. Therapy often involves extracorporeal ultraviolet phototherapy and single-agent cytotoxic chemotherapeutic agents such as methotrexate^4^. However, despite aggressive therapies, initial response rates are poor and disease recurrence is common^5^.

To date, efforts to identify genes recurrently targeted by mutation in SS genomes have been largely targeted^6^,^7^,^8^, or otherwise limited to a few index samples^9^,^10^. The comprehensive genomic landscape of SS has not been explored and opportunities for targeted therapies based on specific genetic mutations have not been fully exploited. To gain insights into the genetic alterations underlying the pathogenesis of SS, we integrated whole-genome sequencing (WGS) and whole-exome sequencing (WES) in combination with high-resolution copy-number variant (CNV) analysis on a large cohort of well-characterized cases of SS. Our studies reveal recurrent mutations targeting epigenetic modifiers and JAK–STAT pathway in SS.

## Results

### WGS reveals genomic complexity of SS

**Figure 1:**
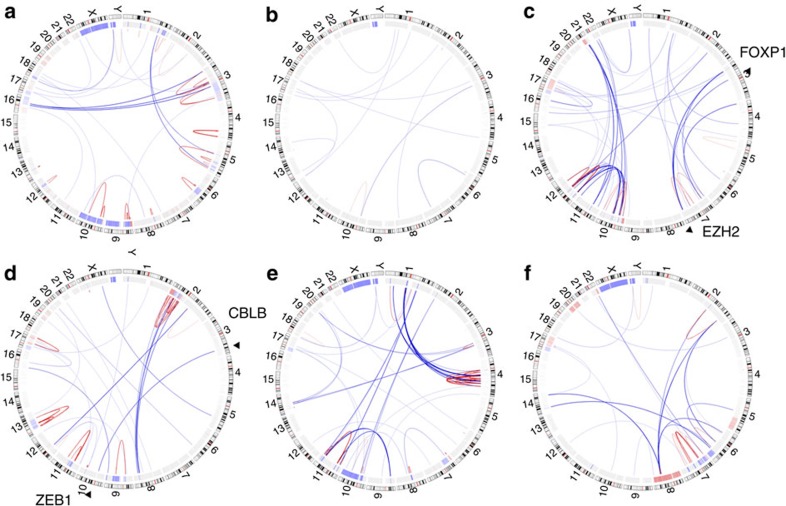
Structural alterations in six Sézary syndrome genomes identified by whole-genome sequencing. Circos diagrams for six SS genomes: panels **a** (case A02), **b** (case B02), **c** (case C02), **d** (case D02), **e** (case G01) and **f** (case H01) depict the chromosomes arranged circularly end to end with each chromosome's cytobands marked in the outer ring. The inner ring displays copy-number data inferred from whole-genome sequencing with blue indicating losses and red indicating gains. Within the circle, rearrangements are shown as arcs with intrachromosomal events in red and interchromosomal translocations in blue. The two representative translocations involving *EZH2-FOXP1* and *ZEB1-CBLB* are highlighted in **c** and **d**, respectively.

To obtain a genome-wide view of the molecular genetic alterations underlying SS at a nucleotide resolution level, we performed WGS of highly enriched (>90%) pure tumour cells from six cases that fulfilled established diagnostic criteria including characteristic cytologic, immunophenotypic and karyotypic features^3^. The data highlight the structural genomic complexity of SS (Fig. 1; comprehensive structural alteration data from WGS can be found in Supplementary Data 1). This analysis revealed a total of 1,010 inter- or intrachromosomal translocations in the six SS genomes (average 168±43 translocations per genome). No recurrent translocations or gene fusions were identified in these six SS cases. However, among 42 potential fusion genes (Supplementary Data 2), several noteworthy candidates were identified that may contribute to SS disease pathogenesis in selected cases.

Novel translocations identified in SS included juxtaposition of the N terminal of receptor tyrosine kinase *TPR* and the C terminal of hepatocyte growth factor receptor *MET* previously identified to be oncogenic in gastric cancers^11^; the N terminus of *v-myb* avian myeloblastosis viral oncogene homologue-like 1 *MYBL1* fused with the C terminal of thymocyte high-mobility group box protein of *TOX* previously implicated in the pathogenesis of mycosis fungoides/SS^12^; and the N terminus of the Hsp40 homologue *DNAJC15* and the C terminus of zinc-finger protein *ZMYM2* involved in the translocation t(8;13)(p11;q12), which fuses *FGFR1* with *ZMYM2* associated with 8p11 myeloproliferative disorders^13^. Other noteworthy translocations separately targeted the *MEIS1* homeobox gene previously implicated in leukemogenesis^14^,^15^, the transcription factor *IKZF2*, and the serine–threonine kinase *VRK2* (Supplementary Data 2). WGS also revealed a novel reciprocal translocation event involving chromosomes 3 and 10 leading to interruption of coding elements of *CBLB* between exons 13 and 14 by insertion of the coding elements of *ZEB1* beginning at exon 2. This translocation is predicted to result in a fusion gene composed of the N-terminal portion of *CBLB* (residues 1–653 including CBL-PTB, UBA, RING EF-hand and SH2 domains) with the entire protein structure (including all seven zinc-fingers and DNA-binding domains) of ZEB1, a zinc-finger negative transcriptional regulator of interleukin-2-stimulated cytokine signalling in T cells^16^. Also of note, a translocation event involving elements of the histone-lysine *N*-methyltransferase *EZH2* on chromosome 7 juxtaposed to elements upstream of the forkhead box protein *FOXP1* on chromosome 3 was observed in one SS genome. In addition to the large structural variations, WGS analyses revealed recurrent aneuploidies in SS genomes including trisomy 8 (1/6 cases) and monosomy 10 (2/6); and loss of 17p and/or isochromosome 17 (5/6). Interestingly, losses of chromosome 1p, reported to be recurrently deleted in several aCGH studies^17^,^18^,^19^,^20^,^21^,^22^, were identified in 4/6 genomes with a narrowly restricted region spanning 1p36.21-1p35.3. To date, no candidate gene within this region has been identified and implicated in SS pathogenesis.

### CNV and WES analyses reveal recurrent loss-of-function loci

To identify regions of recurrent gain or loss at high resolution and thereby define genes targeted by numerical aberrations, we performed high-resolution aCGH on a total of 80 SS samples (Supplementary Fig. 1). This analysis revealed a number of recurrent gains (Supplementary Fig. 2) and losses (Supplementary Fig. 3) of chromosomal material (comprehensive CNV data is presented in Supplementary Data 3). These disruptions included previously reported alterations such as recurrent gains of chromosome 8 (∼25%) and loss of the short arm of chromosome 17 (∼40%), which frequently also involved simultaneous gains of the long arm of chromosome 17 (∼25%) of SS cases (Supplementary Fig. 4). A high-frequency of deletions of the short arm of chromosome 19 was observed. The data also defined specific regions of recurrent aneuploidy at chromosome 1p36.11, 3p21.31, 9p21.3, 10p11.22 and 13q14.2 (Supplementary Fig. 3, arrowheads). To precisely identify single-gene candidates within these chromosomal loci that may contribute to SS pathogenesis through gene mutation, we performed WES on 60 additional SS genomes (comprehensive mutational data presented in Supplementary Data 4).

Our WES data demonstrated that genes within regions of recurrent deletions previously demonstrated to be important in SS pathogenesis (*TP53* on chromosome 17, *CDKN2A* on chromosome 9 and *PTEN* on chromosome 10) also were affected by deleterious mutations. Specifically, WES identified loss-of-function mutations in CDKN2A (p.W15X nonsense mutation), a frameshift mutation in *PTEN* in the C2 functional domain and numerous nonsense, frameshift and splice junction mutations in *TP53* (Supplementary Fig. 5). To efficiently identify novel genes contributing to the pathogenesis of SS, we selected those genes which showed both a high rate of deletion (>10% of 80 SS genomes) as well as genes with at least one known deleterious mutation (either frameshift, nonsense and/or splice donor or acceptor mutation among the 66 SS genomes sequenced). This approach revealed several candidate genes involved in the pathogenesis of SS.

### Loss of function of ARID and SMARC family proteins

**Figure 2:**
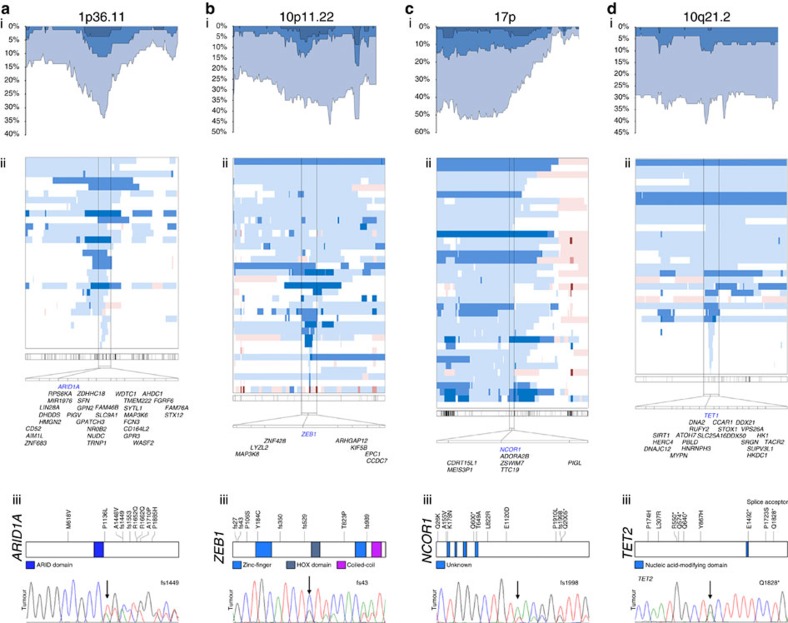
Recurrent loss-of-function alterations of chromatin remodelers and DNA modifier genes. Evidence of loss of function in Sézary syndrome (SS) genomes including (i) proportion of total genome locus affected, (ii) depiction of individual genomes of SS with very narrowly defined deletions specifically targeting the highlighted gene and (iii) deleterious mutations including frameshift and missense mutations confirmed to be somatic by Sanger sequencing for (**a**) *ARID1A*, (**b**) *ZEB1*, (**c**) *NCOR1* and (**d**) *TET2*.

**Figure 3:**
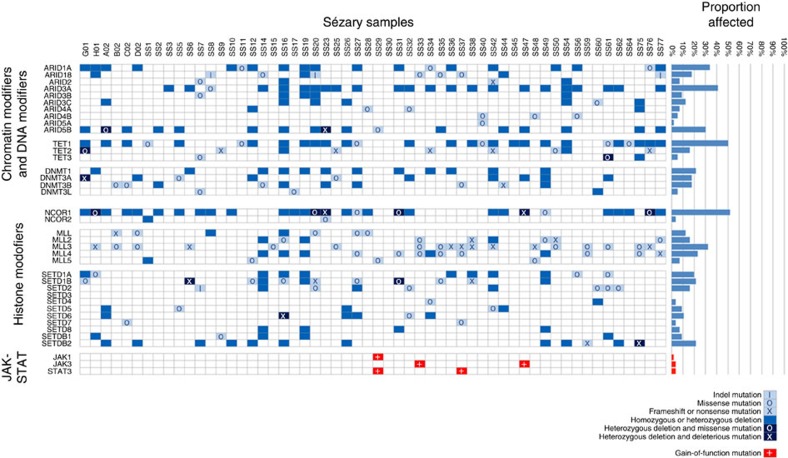
Epigenetic modifiers and JAK–STAT pathway components are targeted by aneuploidy and mutations. Summary of deletions and/or mutations in SS genomes affecting genes involved in epigenetic modifications. The loss-of-function of epigenetic modifiers including *ARID* family members, *TET*s, *DNMTs*, *NCOR1*, *MLLs* and *SET* domain containing genes are indicated in blue while gain-of-function mutations in *JAK* and *STAT* family members are indicated in red.

Examination of the most frequently restricted region of recurrent deletion, the 1p36.11 locus, revealed 18 genes with at least one deleterious mutation (Supplementary Data 5). Of these, *ARID1A* exhibited the highest frequency of disruption and/or mutation with 27/80 genomes involved by deletions and 7/66 showing novel mutations including a frameshift mutation at residue 1449 within the GR-binding domain where the mutations seemed to co-localize; Figs 2a and 3; Supplementary Fig. 6). Disruption of *ARID1A* by either deletional events or mutation or both were seen in 25/62 (40.3%) SS genomes on which both WES and aCGH analyses were performed. Loss-of-function mutations in *ARID1A* have been implicated in the pathogenesis of other hematopoietic and non-haematopoietic malignancies^23^, but not in SS.

Another member of the ARID family of chromatin remodelling genes, *ARID5B*, was also implicated as a candidate because deletions of region 10q21.2 were observed in 23/80 genomes and point mutations in 3/66 SS genomes. The latter group included a nonsense mutation p.K239X (Fig. 3; Supplementary Fig. 7. *ARID5B* is recurrently mutated in endometrial malignancies^24^ and deleted in a fraction of paediatric B-cell acute lymphoblastic leukaemia^25^.

ARID family members play a critical role in the SWI/SNF complex where they exhibit helicase and ATPase activities and regulate transcription of genes by altering the surrounding chromatin structure^26^. SWI/SNF family members, including the *SMARC* group, interact with proteins encoded by the *ARID* family member genes^27^. Loss of SWI/SNF complex member SNF5 leads to T-cell lymphoma at high penetrance levels^28^. Interestingly, our studies revealed that *SMARCC1* at 3p21.31 was recurrently targeted by deletions (<4 Mb) in 17/80 (21.3%) of SS genomes (Supplementary Fig. 3). Strikingly, deletions and/or mutations in either *ARID1A*, *ARID5B* or *SMARCC1* were identified in 38/62 (61.3%) of SS genomes in which both WES and aCGH were performed. WES revealed mutations in other members of the ARID and SMARC family, namely, *ARID1B*, *ARID4A* and *ARID2*, which also included a case with a nonsense mutation (ARID2 p.Q1462X). Moreover, *ARID3A* and *SMARCA4*, recently found to be mutated in small cell carcinoma of the ovary, are both located on the short arm of chromosome 19, which is recurrently deleted in 29/80 and 18/80 SS genomes, respectively. Altogether, deletions and/or mutations of at least one of the 10 *ARID* or 13 *SMARC* family genes were identified in 91.9% of SS genomes (Fig. 3).

*ZEB1*, in addition to being implicated in a fusion event involving *CBLB* as described above (Fig. 1d; Table 1), was also recurrently targeted by deletions of chromosomal material (29/80 SS genomes) as well as deleterious mutations (7/66 SS genomes) including five frameshift mutations in four SS genomes (Fig. 2b). In total, 28/62 (45.2%) of SS genomes showed evidence of loss-of-function alterations of *ZEB1* strongly implicating this gene as a tumour suppressor involved in the pathogenesis of SS. This concept is supported by the demonstration that TCF8/*ZEB1* mutant mice frequently develop a CD4-positive T-cell lymphoma/leukaemia with a median onset of 30 weeks^29^,^30^. Further, *ZEB1* has been implicated as a candidate tumour suppressor gene in adult T-cell leukaemia/lymphoma^30^. ZEB1 directly interacts with the trithorax group protein component SMARCA4 (also known as BRG-1) and recruits histone deacetylases HDAC1 and HDAC2 (refs 16, 31). Our results also showed that the cyclic AMP-response element-binding protein-binding protein, which is frequently mutated in acute lymphoblastic leukaemia^32^ and critical for trithorax group protein activity^33^, was recurrently altered by deletions in 19/80 and mutations in 7/66 SS genomes including a p.Q1108X nonsense mutation and several frameshift mutations (Supplementary Data 4).

### MLL and SETDB and KDM6B mutations

The results also implicated members of the trithorax group histone methyltransferases, particularly *MLL2*, known to be frequently mutated in a variety of human malignancies^34^,^35^,^36^. These genes were deleted in 11 of 80 SS genomes. *MLL2* was targeted in 7/66 SS genomes including one with a monoallelic nonsense mutation (p.Q4219X) and another genome with bialleleic nonsense mutations (p.S624X and p.S687X). *MLL4* was mutated in 12/66 of SS genomes including one frameshift mutation (p.P622fs; Fig. 3; Table 1). In addition, *MLL3* was targeted in 39/66 SS genomes, including four genomes with frameshift or nonsense mutations (Fig. 3). SETD family histone methyltransferases were involved by recurrent alterations including *SETD1A* deletions in 14/80 SS genomes and mutations in 4/66 genomes including one frameshift (Fig. 3; Supplementary Fig. 8); *SETD1B* deletions in 12/80 and mutations in 11/66 SS genomes including one frameshift and one nonsense mutation (p.E66X; Fig. 3; Supplementary Fig. 9); *SETDB2* deletions in 18/80 and mutations in 2/66 SS genomes including two nonsense mutations (p.R588X) in separate genomes; and *SETD6* deletions in 9/80 and mutations in 2/66 SS genomes including one frameshift mutation (Fig. 3). Altogether, deletions and/or mutations in *MLL, MLL2, MLL3, MLL4, SETD1A, SETD1B, SETDB2* and *SETD6* were identified in 67.7% of SS genomes. Moreover, deletions of the histone-lysine demethylase KDM6B on chromosome 17p were observed in 38/80 SS genome, whereas mutations were seen in 11/66 SS genome including one frameshift mutation p.P471fs.

In addition, we identified genetic alterations affecting other epigenetic modulators including the histone deacetylase *NCOR1* on the short arm of chromosome 17 including deletions in 38/80 and mutations in 9/66 including one frameshift mutation (p.Y1997fs) and two nonsense mutations (p.Q600X and p.Q2005X; Figs 2c and 3). *NCOR1* is implicated in the pathogenesis of acute lymphoid leukaemia and non-haematopoietic malignancies^37^,^38^. Six of 62 SS genomes showed simultaneous mutation and deletion events suggesting biallelic loss of function of *NCOR1* (Fig. 3). These results indicate that disruption of homeostatic histone modification may contribute to the pathogenesis of SS.

Further implicating a role for deregulation of epigenetic modifiers in SS was our identification of alterations targeting multiple members of the DNA methyltransferase family, including *DNMT3A*, which has been implicated in the development of acute myeloid leukaemia^39^. *DNMT3A* was deleted in 14/80 and mutated in 5/66 SS genomes, including the novel nonsense mutation p.Y394X. In addition, *DNMT3B* was deleted in 9/80 and mutated in 5/66 SS genomes including the nonsense mutation p.R223X (Fig. 3). Additional genes involved in DNA methylation such as the demethylases, *TET1* and *TET2*, recently implicated in the pathogenesis of myeloid malignancies^40^,^41^, also showed evidence of loss-of-function alterations. Strikingly, among the SS genomes showing deletion of any portion of chromosome 10 were eight cases with narrowly focused deletions (<0.5 Mb) involving the *TET1* gene. Moreover, sequencing identified recurrent mutations affecting *TET2* in 7/66 SS genomes including five nonsense mutations and a disrupted splice acceptor mutation (Fig. 2diii). Altogether, deletions and/or mutations in *DNMT3A, DNMT3B, TET1* or *TET2* were identified in 74.2% of SS genomes (Fig. 3). Taken together, these results support a critical role for deregulated DNA methylation in the pathogenesis of SS.

### Loss-of-function alterations in cell cycle regulators

Our integrated approach highlighted involvement in SS of several novel candidate gene families involved in cell cycle regulation whose members were recurrently altered through both deletions and mutations. Loss of the well-known tumour suppressor gene *CDKN1B* on 12p13.1 was identified in 12/80 SS genomes, including a case showing a deletion and a single p.W76X nonsense mutation. In addition, the *RB1* gene locus at 13q14.2 was deleted in 21/80 SS genomes with a number of narrowly restricted deletions (<0.7 Mb; Supplementary Fig. 5).

### Gain-of-function mutations target PLCG1 and CARD11

PLCG1 is an important mediator in the T-cell receptor/CD3-NFAT signalling axis critical for T-cell activation and differentiation^42^.We identified recurrent gain-of-function mutations in *PLCG1* including p.S345F (cases SS26 and SS31) affecting the PLCx catalytic domain and p.S520F (case SS84). In addition, we identified three additional mutations p.R1158H (case SSG01), p.R1163K (case SS9) and p.VYEEDM1161V (case SS86) tightly clustered in the C2 domain, which is a calcium-dependent membrane targeting module involved in signal transduction (Supplementary Fig. 10a).

The multidomain scaffold protein CARMA encoded by the *CARD11* gene is a positive mediator for the NF-κB activation involved in antigen receptor signalling^43^. We identified recurrent missense mutations in *CARD11* including p.M360K (case SS12), p.V266A (case SS26), p.D357A (case SS42) and p.S618F (cases SS26 and SS32; Supplementary Fig. 10b).

Overall, 6/66 (9.1%) harboured *PLCG1* mutations and 4/66 (6%) harboured *CARD11* mutations. These findings suggest a role for antigen receptor signalling mediators in the pathogenesis of SS.

### Mutations in JAK–STAT and Ras pathways

**Figure 4:**
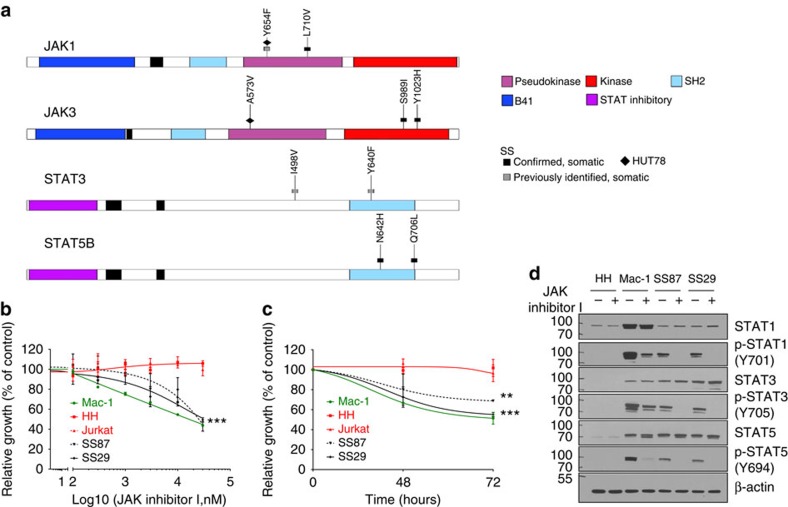
Validation of gain-of-function of JAK–STAT pathway in Sézary syndrome (SS). (**a**) Schematic representations of mutations in *JAK1, JAK3, STAT3 and STAT5B* identified in primary SS samples and the Hut-78 SS-derived cell line. (**b**) Dose–response curves for two primary SS leukaemic cells (SS29 and SS87), the negative control cell lines (HH and Jurkat) and the positive control cell line (Mac-1) after 48 h of treatment with JAK inhibitor I. All experiments were performed in triplicate and error bars represent s.d's (***P<0.001, *T*-test). (**c**) Time–response curve for the two primary Sézary syndrome leukaemic cells (SS29 and SS87), the negative control cell lines (HH and Jurkat) and the positive control cell line (Mac-1) treated with 3 μM of JAK inhibitor I. All experiments were performed in triplicate and error bars represent s.d.'s (**P<0.01 and ***P<0.001 *T*-test). (**d**) Phosphorylation of STAT1, STAT3 and STAT5 in the two primary SS leukaemic cells (SS29 and SS87), the negative control cell lines (HH and Jurkat) and the positive control cell line (Mac-1) treated with 3 μM of JAK inhibitor I for 4 h.

WES revealed somatic gain-of-function mutations affecting *JAK1* (2/66, 3%), *JAK3* (2/66), *STAT3* (2/66) and *STAT5B* (2/66) altogether present in 11% of cases (Fig. 3; Table 2). Importantly, recurrent mutations of *JAK1, JAK3, STAT3* and *STAT5B* have not been previously reported in SS and were corroborated by Sanger sequencing in all cases (Fig. 4a). The mutations were clustered in the autoinhibitory pseudokinase domain and included variants detected previously in other malignancies or shown to lead to constitutive activation of JAK–STAT^44^ signalling including JAK1 p.Y654F^45^,^46^ and p.L710V. By comparison, the JAK3 mutations p.S989I and p.Y1023H were located in the kinase domain. This observation is intriguing given the recent observation that some mutations in the kinase domain of JAK3 confer interleukin-3 independence to Ba/F3 cells *in vitro*^47^. By WES we identified known gain-of-function mutations p.I498V^48^ (Table 2) and p.Y640F^49^ (Table 2) in *STAT3*, as well as p.N642H and p.Q706L (2/66, 3%) in *STAT5B* in SS. The JAK–STAT pathway mutations were mutually exclusive in all but one case (SS29) that harboured both *STAT3* and *JAK1* mutations. Matched constitutional DNA from normal tissue (for example, CD4-negative leukocytes from peripheral blood) confirmed somatic acquisition of these mutations (Fig. 4a; Table 2). Of additional interest, we identified mutations affecting the *Ras* homologue gene family member *RhoA* in 4/66 (6%) cases, namely, p.C16R (case SS8), p.N117K (cases SS33 and SS60) and p.K118T (case SS47). We also observed a known oncogenic mutation affecting NRAS (p.Q61K) in the SS-derived cell line Hut-78. Taken together, these mutations implicate a role for mutations affecting the JAK–STAT and RAS family pathways in the pathogenesis of SS.

### JAK-mutated primary SS cells are sensitive to JAK inhibitors

To investigate the possible utility of targeting the JAK–STAT pathway, two primary SS samples harbouring mutations in *JAK1* (SS87 p.Y654F and SS29 pL710V) were subjected to JAK inhibition. Exposure to increasing doses of JAK inhibitor I^50^ led to substantial reduction in cell proliferation of primary SS cells (Fig. 4b,c). Western blotting studies revealed inhibition of phosphorylation and activation of STAT1, STAT3 and STAT5 signalling in the SS cells harbouring both JAK1 mutations, whereas the mature T-cell lymphoma-derived cell line, HH, which does not carry mutations in the JAK–STAT axis, did not show any changes (Fig. 4d; Supplementary Fig. 12). Similarly, the primary SS cells harbouring the JAK1 mutations and the SS-derived cell line Hut-78 carrying the JAK1 p.Y654F and JAK3 p.A573V mutations were sensitive to the JAK1/2-selective inhibitor ruxolitinib. On the other hand, the JAK mutation-negative mycosis fungoides/SS-derived cell line HH and the JAK mutation-negative T-cell line Jurkat were both unresponsive to ruxolitinib treatment (Supplementary Fig. 11). Taken together, these data indicate that pharmacological antagonism of JAK1–STAT signalling inhibits growth of primary SS tumour cells. The effects of these mutations and of pharmacologic targeting of the *JAK1*–*STAT* pathway are diagrammed in Fig. 4.

## Discussion

This study provides for the first time a comprehensive genomic landscape of SS. The integrated genomic analysis of 80 SS genomes we present reveals a complex genomic landscape with numerous structural variations including translocations, loss-of-function deletions and mutations as well as actionable gain-of-function mutations. WGS showed numerous (*n*=42) fusion genes including *TPR-MET*, *MYBL1-TOX*, *DNAJC15-ZMYM2* and *EZH2-FOXP1* which, albeit not recurrent, could contribute to the pathogenesis of SS. The WGS analyses also confirms previously observed recurrent aneuploidies, including trisomy 8, monosomy 10 and loss of 17p and/or isochromosome 17 indicating that chromosomal instability is a characteristic of this malignancy and may contribute to the poor clinical behaviour.

The data derived from this integrated genomic analyses of the SS genome show a striking profile of recurrent loss-of-function alterations in the form of deletions and inactivating mutations targeting the epigenetic modification machinery including the *ARID/SMARC* family chromatin remodelling complex, histone methyltransferases (*MLLs* and *SETD1A/B*) and demethylase (*KDM6B*), DNA methyltransferase family members and *TET1/2/3* genes. These results highlight the substantial contribution of epigenetic deregulation in the pathogenesis of SS and provide targeted insights to study the mechanisms of sensitivity and resistance to inhibitors of histone deacetylases and DNA methyltransferases in SS patients. Recurrent loss-of-function alterations targeting the well-characterized tumour suppressor genes *TP53, CDKN2A*, *PTEN* as well as cell cycle regulators *CDKN1B* and *RB1* were also common in SS.

Dysregulation of T-cell receptor signalling has been implicated in the pathogenesis of T-cell neoplasia^51^. Our studies reveal that mutational subversion of components of signalling pathways critical for T-cell activation and survival is common in SS. In this regard, recurrent somatic gain-of-function mutations involving *PLCG1*, *JAK1/3* and *STAT3/5B* are a prominent feature of the SS mutational landscape. Inhibition of activated pathways, particularly those driven by oncogenic tyrosine kinases, has served as a molecularly tractable strategy for the targeted therapy of cancers. In this regard, we demonstrated that pharmacologic antagonism of JAK in primary SS samples harbouring *JAK1* mutations led to significant inhibition of tumour cell proliferation.

In conclusion, the genomic landscape of SS reveals a heterogeneous genetic portrait with frequent disruption of epigenetic modifiers, cell cycle regulators and subversion of signalling modules involved in T-cell receptor, chemokine and cytokine signalling pathways critical for T-cell activation, survival and differentiation. Overall, these studies highlight the opportunities afforded by integrated genomic approaches in elucidating the molecular genetic alterations underlying the pathogenesis of SS. We anticipate that the findings presented here will play a role in refining disease taxonomy and offer opportunities for the definition of personalized biomarkers as well as precision therapeutics for patients with SS.

## Methods

### Patients and samples

Clinical samples were obtained from the archives of the Department of Pathology at The University of Texas MD Anderson Cancer Center, the University of Michigan and The University of Utah Health Sciences Center. Institutional Review Board approval for access to clinical specimens and information was obtained from the University of Michigan, the University of Texas MD Anderson Cancer Center and the University of Utah. Informed consent was obtained from the patients for use of diagnostic material. All SS cases fulfilled pathologic criteria for diagnosis of SS according to World Health Organization classification criteria. For a given patient, samples represented either formalin-fixed, paraffin-embedded tissue (FFPE), cryopreserved peripheral blood leukocytes or both. Where applicable, constitutional normal tissue represented either tumour-free FFPE tissue derived from the same patient or otherwise tumour-depleted peripheral blood leukocytes generated using EasySep column enrichment and B220- and/or Mac-1-positive cell selection (Stem Cell Technologies, Inc.). Relative tumour- depletion of resultant cell suspensions was determined by flow cytometry using an antibody directed against CD4 (BD Pharmingen). DNA was extracted from both FFPE and frozen samples using QIAGEN DNA extraction kits according to manufacturer's instructions.

### aCGH analysis

CNVs were detected using Nimblegen whole-genome arrays containing 270,000 features (Roche Applied Science) sufficient to detect CNVs of 50 kb or greater. Arrays were prepared according to manufacturer's protocol and analysed on NimbleGen MS 200 Scanner followed by data extraction, normalization and processing for segMNT analysis using NimbleScan software according to manufacturer's instructions. Downstream data processing and interpretation were performed using custom-designed processing algorithms solely reliant on the data comprising output segMNT data files. Circos plots were generated to display the segMNT data. The aCGH data are available at http://www.ncbi.nlm.nih.gov/bioproject/PRJNA292537 (index cases) and http://www.ncbi.nlm.nih.gov/bioproject/PRJNA292547 (discovery cases).

### Sequencing

For WGS, 7–10 μg of high-molecular-weight genomic DNA was extracted from fresh frozen tumour tissue and subjected to WGS by Complete Genomics, Inc. (CGI; Mountain View, CA). CGI performs massively parallel short-read sequencing using a combinatorial probe-anchor ligation chemistry coupled with a patterned nanoarray-based platform of self-assembling DNA nanoballs. (Library generation, read-mapping to the NCBI reference genome (Build 37, RefSeq Accession nos. CM000663-CM00686). Initial read mapping and variant calling were performed using CGAtools v1.3.0 (http://cgatools.sourceforge.net/docs/1.3.0/). Additional downstream bioinformatic analyses of WGS data were performed using custom-designed processing routines. WGS yielded a mean of 351±13 Gb mapped per sample with 97.4–97.8% fully called genome fraction and 97.1–97.7% fully called exome fraction. The median genomic sequencing depth exceeded 60 × in all samples normalized across the entire genome.

For WES, genomic DNA samples were fragmented using a Covaris S2 fragmentation system to a target size of 400 bp. The samples were end-repaired, a-tailed and custom adapters were ligated using the NEBNext DNA Library Prep kit according to the manufacturers recommended protocols. The custom adapters included 6-bp barcodes and were synthesized by Integrated DNA Technologies. After ligation, the samples were size selected to 400 bp on a 2% agarose gel and 1-mm gel slices were retained. Samples were isolated from the gel using the Qiagen QIAquick gel extraction system. Seven microlitres of each ligation product was enriched using the Phusion master mix kit and custom PCR primers with a total of 14 cycles of PCR amplification (TruSeq Universal Adapter 5′-AAT-GAT-ACG-GCG-ACC-ACC-GAG-ATC-TAC-ACT-CTT-TCC-CTA-CAC-GAC-GCT-CTT-CCG-ATC-T-3′ and TruSeq Adapter, Index 1 6 5′-GAT-CGG-AAG-AGC-ACA-CGT-CTG-AAC-TCC-AGT-CAC-ATC-ACG-XXX-XXX-TAT-GCC-GTC-TTC-TGC-TTG-3′). Two PCR amplifications were performed for each sample. The PCR products were pooled and purified using AmpureXP beads.

Library QC was performed using the Agilent Tapestation and qPCR. On the basis of qPCR concentrations, 200 ng of each of five samples were pooled for a total of three pools. Each pool was captured using the Nimblegen SeqCap EZ V3 Exome Enrichment Kit according to the manufacturer's recommended protocols. The capture pools were combined and sequenced on the Illumina HiSeq 2,000 platform across four lanes with paired-end 100-bp reads. The average depth of coverage for exome sequencing was 30.4±11.9 × with >95% of coding exons sequenced to a depth of 30 or more.

Our analysis focused on variations that were not in the Database of single-nucleotide proteins (dbSNP) and/or those that had been associated previously with cancer based on information in the Catalogue of Online Somatic Mutations in Cancer (COSMIC) database. After identifying candidate mutations of interest, 10–50 ng of genomic DNA from tumour and matched constitutional tissue or otherwise tumour-depleted, matched normal DNA was subjected to Sanger sequencing. To confirm somatic acquisition, tumour-depleted or tumour-free matched normal DNA was also subjected to WES to identify somatic alterations in SS (CD4-negative cells obtained following immunomagnetic enrichment of CD4-positive SS cells or skin uninvolved by tumour). Three parameters were combined to arrive at threshold criteria for designation of a variant as somatic in the absence of constitutional information. First, all of the variants identified in the present study were filtered to exclude variants in the dbSNP database. To develop a parameter for allele burden based on read counts obtained from next-generation sequencing, we leveraged our NGS database containing NGS data from >500 genomes and modelled the allele frequency correlation between germline variants and their corresponding read counts. Depending on the read depth, the allele frequencies of germline variants varied from 40 to 60% (variant/wild-type ratio). On the other hand, variants exhibiting ≤25 or ≥75% variant/wild-type ratios consistently and invariably represented somatic alterations. Thus, we employed these thresholds as cutoffs for designation of somatic variants. In addition, Sanger sequencing was performed to validate findings observed for 75 variants and qualitatively and quantitatively corroborated the results of NGS. Accordingly, in scenarios where constitutional DNA was not available, we assessed the allele burdens using read counts and those with mutant-to-wild-type ratios <25 or >75% were subjected to Sanger sequencing to corroborate the ratio from peak heights and scored as mutant when these ratios were maintained. Finally, when all parameters (that is, allele burden by NGS confirmed by Sanger and excluded by dbSNP) were fulfilled, the variants were presumed to be somatic. For all sequencing reactions, PCR amplification was performed using Phusion DNA polymerase (New England Biolabs) followed by conventional Sanger sequencing technology using BigDye version 3.1 chemistry run on an Applied Biosystems 3730 × l DNA Sequencer at the University of Michigan DNA sequencing Core. All sequencing reactions were performed using nested sequencing primers. Sequencing trace analysis was performed using Mutation Surveyor software. All primers were designed using a custom-developed program and purchased from Integrated DNA Technologies lyophilized in 96-well plates.

WES FASTQ sequencing data files were aligned to human NCBI Build 36 reference sequence using BWA 0.6.2 available at http://bio-bwa.sourceforge.net/. Merging and deduplication were performed using Picard 1.79 available at http://broadinstitute.github.io/picard/. The DepthOfCoverage, CountReads, RealignerTargetCreator, IndelRealigner, BaseRecalibrator, PrintReads and UnifiedGenotyper functions within GenomeAnalysisTK-1.6-9 (available at https://www.broadinstitute.org/gatk/) were used to ascertain coverage and for variant calling. Resultant VCF files were annotated using snpEff v3.1 available at http://snpeff.sourceforge.net/. Variants previously identified as somatic in malignancy were highlighted if present in the COSMIC database (http://cancer.sanger.ac.uk/cosmic). Additional custom in-house bioinformatics pipelines were designed to interpret, collate, organize and display the integrated results obtained from next-generation sequencing and aCGH data open source software tools described above will be published in a subsequent manuscript and are as yet not publically available. Custom-designed data processing algorithms used to process aCGH data for display and interpretation and for automated primer design will likely not be published and will not be made publically available as numerous open source and commercially available tools exist for these analyses. The sequencing data are available at http://www.ncbi.nlm.nih.gov/bioproject/PRJNA292537 (index cases) and http://www.ncbi.nlm.nih.gov/bioproject/PRJNA292547 (discovery cases).

### Cell lines

Jurkat, HH and Hut-78 cell lines were obtained from the American Type Culture Collection. Mac-1 was kindly provided by Dr Marshall E. Kardin (Boston University School of Medicine, Massachusetts). All cell lines were grown in RPMI-1644 media supplemented with 10% fetal bovine serum (FBS) except Hut-78, which was grown in IMDM media supplemented with 10% FBS. No mycoplasma contamination was detected at the time of experiments.

### Primary cell culture and JAK inhibitor treatment

Primary tumour cells were isolated from peripheral blood of SS patients for routine diagnosis and excess aliquots viably cryopreserved in FBS/dimethylsulphoxide at −140 °C. All samples were obtained with IRB approval from the University of Michigan and consents were obtained. Primary SS cells harbouring *JAK1* gain-of-function mutation were thawed and grown overnight in RPMI cell culture medium followed by treatment with JAK inhibitor I (EMD Millipore), a pan inhibitor of JAK kinase activation, at different concentrations as indicated. A measure of 3 μM of JAK inhibitor I was used for time kinetics of cell growth assay on primary SS cells as indicated. Cell growth was determinate at 48 and 72 h in triplicate by WST-1 (Roche Diagnostics) according to the manufacturer's instructions. Each assay was performed in triplicate.

### Immunofluorescence

For immunofluorescence studies, cells were plated in chamber slides. Cells were fixed in 4% formaldehyde, permeabilized with 0.5% Triton X-100 for 5 min and coimmunostained with anti-ARID1A (clone 3H14, MP Biomedicals, 1/1,000) followed by staining with Alexa Fluor 594-conjugated secondary antibodies (Molecular Probes, Eugene, OR). The slides were mounted with Vectashield mounting medium containing 4′,6-diamidino-2-phenylindole (Vector Laboratories, Burlingame, CA). After staining, cells were photographed (× 40) using a Nikon Eclipse 80i confocal microscope.

### Western blot

For western blot analysis, cells were treated for 4 h with JAK inhibitor I at 3 μM and protein lysates were subjected to western blot analysis. The following primary antibodies were obtained from Cell Signaling Technology: STAT1 (clone 9H2, 1/1,000), p-STAT1 (clone 58D6, 1/1,000), STAT3 (clone 79D7, 1/1,000), p-STAT3 (clone M9C6, 1/1,000), STAT5 (polyclonal, 1/1,000) and p-STAT5 (clone D47E7, 1/1,000). The loading quality was assessed using antibody against β-actin (clone AC-74, 1/10,000) from Sigma-Aldrich.

## Additional information

**How to cite this article:** Kiel, M. J. *et al*. Genomic analyses reveal recurrent mutations in epigenetic modifiers and the JAK–STAT pathway in Sézary syndrome. *Nat. Commun.* 6:8470 doi: 10.1038/ncomms9470 (2015).

## Supplementary Material

Supplementary Information Supplementary Figures 1-12

Supplementary Data 1 Comprehensive list of structural alterations in 6 index SS cases. Listed are the structural alterations for each of 6 index Sezary Syndrome genomes with the partner genomic regions identified by chromosome, position and strandedness. Left partner is arbitrarily-defined as the first of the two junctional positions within the genome. The distance, strand consistency and whether the alteration is strand consistent are listed as are the number of discordant reads supporting each alteration. If alteration affects one or more genes, the gene symbols and transcripts are listed as is the final assembled junctional sequence.

Supplementary Data 2 List of potential fusion genes in 6 index SS genomes The subset of structural alterations listed in Supplementary Data 1 for which a viable fusion-gene might result based on alteration strandedness and gene strandedness are detailed.

Supplementary Data 3 Comprehensive list of copy number variations in SS cases. The copy number alterations identified by array-CGH assays are detailed with red representing gain of chromosomal material and blue representing loss. The darker colors represent greater gains (more than 1.5x gains) or losses (less than 0.5x losses) at each of the specified loci for each of the 80 genomes for which there was sufficient DNA to perform aCGH. Shown at the far right are the genes (based on earliest chromosomal position) contained within each locus.

Supplementary Data 4 Comprehensive list of novel gene mutations identified in SS cases. Detailed information of all novel (defined as not being present in dbSNP) mutations identified in each of 66 Sezary Syndrome genomes itemized according to individual genes. Multiple mutations for a given gene in a single patient are separated by the "|" symbol. Frameshift mutations are highlighted in purple, non-sense mutations in red and mutations previously described in the COSMIC database (see Methods) in blue. Missense mutations are displayed in green. Other details for each include the protein coding consequence of the change as well as the chromosome, position and reference and alternate alleles for each mutation.

Supplementary Data 5 Truncated list of genes showing more than 10% of SS genomes with deletions and at least one deletorious mutation. This table combines the data contained within Supplementary Data 3 and 4 for those genes with at least 10% of Sezary Syndrome genomes showing deletions by aCGH and at least one deleterious mutation (defined as frameshift or nonsense mutations).

## Figures and Tables

**Table 1:** Recurrent gene mutations in DNA and histone modifiers.

**Gene**	**ID**	**Consequence**	**Amino-acid change**	**Chr**	**Position**	**Ref**	**Alt**	Somatic acquisition confirmed*
*ARID1A*	SS34	Missense	p.M618V	1	27,059,215	A	G	Yes
	SS18	Missense	p.P1163L	1	27,099,072	C	T	Yes
	SS57	Missense	p.A1710P	1	27,106,168	G	C	ND
	SS76	Missense	p.R1652Q	1	27,105,995	G	A	ND
	SS11	Missense	p.R1662Q	1	27,106,025	G	A	ND
	SS84	Frameshift	p.A1448fs	1	27,101,062	A	AT	Yes
	SS84	Missense	p.P1885H	1	27,106,694	C	A	Yes
*ARID5B*	SS23	Nonsense	p.K239X	10	63,850,666	A	T	Yes
	SS29	Missense	p.A375T	10	63,851,074	G	A	ND
	SS29	Missense	p.V558A	10	63,851,624	T	C	ND
	SSA02	Missense	p.R505W	10	63,851,464	C	T	Yes
*DNMT3A*	SS5	Missense	p.G518D	2	25,463,562	C	T	Yes
	SSG01	Nonsense	p.Y395X	2	25,467,123	G	T	ND
*DNMT3B*	SS37	Missense	p.R744S	20	31,393,204	G	T	ND
	SS44	Nonsense	p.R223X	20	31,379,488	C	T	ND
	SS14	Missense	p.A288T	20	31,383,238	G	A	ND
	SSC02	Missense	p.R435C	20	31,386,366	C	T	ND
*MLL2*	SS16	Missense	p.S1762T	12	49,437,685	C	G	ND
	SS33	Missense	p.D2769N	12	49,433,066	C	T	Yes
	SS38	Nonsense	p.Q4219X	12	49,425,833	G	A	Yes
	SS49	Missense	p.V109A	12	49,448,385	A	G	ND
	SS50	Nonsense	p.S687X	12	49,445,406	G	C	Yes
	SS50	Nonsense	p.S624X	12	49,445,595	G	C	Yes
	SS77	Missense	p.A2925V	12	49,432,365	G	A	ND
	SS77	Missense	p.L2245V	12	49,434,820	G	C	ND
*NCOR1*	SS20	Missense	p.P1910L	17	15,961,351	G	A	Yes
	SS23	Nonsense	p.Q600X	17	16,012,157	G	A	Yes
	SS27	Missense	p.T649A	17	16,005,030	T	C	Yes
	SS31	Missense	p.Q26K	17	16,097,808	G	T	Yes
	SS47	Nonsense	p.Q2005	17	15,960,898	G	A	Yes
	SS76	Missense	p.E1120D	17	15,983,762	C	G	Yes
	SS86	Frameshift	p.Y1997fs	17	15,960,917	G	GT	Yes
	SSH01	Missense	p.K178N	17	16,068,377	C	G	ND
*SETD1A*	SS58	Missense	p.P611L	16	30,977,034	C	T	Yes
	SS61	Missense	p.R404H	16	30,976,274	G	A	ND
	SSH01	Missense	p.P595S	16	30,976,985	C	T	ND
*SETD1B*	SS18	Missense	p.P842L	12	122,252,646	C	T	Yes
	SS27	Missense	p.D920E	12	122,254,983	C	G	Yes
	SS31	Missense	p.P353L	12	122,247,909	C	T	ND
	SSG01	Missense	p.P383L	12	122,247,999	C	T	Yes
*SETD6*	SS16	Frameshift	p.M263fs	16	58,552,025	T	TA	Yes
*TET1*	SS40	Missense	p.T87S	10	70,332,355	C	G	ND
	SS52	Missense	p.T1421A	10	70,406,747	A	G	ND
	SS52	Missense	p.D139N	10	70,332,510	G	A	ND
	SS1	Missense	p.Q683E	10	70,404,533	C	G	Yes
*TET2*	SS34	Nonsense	p.E1492X	4	106,194,012	G	T	Yes
	SS42	Nonsense	p.Q640X	4	106,157,017	C	T	Yes
	SS50	Missense	p.P174H	4	106,155,620	C	A	Yes
	SS76	Nonsense	p.Q1828X	4	106,197,149	C	T	Yes
	SS9	Nonsense	p.R550	4	106,156,747	C	T	Yes
	SSG01	Missense	p.L307R	4	106,156,019	T	G	Yes
*ZEB1*	SSC02	Frameshift	p.E42fs	10	31,750,037	GAAGAAAGTGTTAC	G	Yes
	SS50	Missense	p.P106S	10	31,799,636	C	T	ND
	SS19	Missense	p.Y184C	10	31,803,598	A	G	Yes
	SS61	Frameshift	p.L349fs	10	31,809,511	T	TA	Yes
	SS42	Frameshift	p.L528fs	10	31,810,050	TA	T	Yes
	SS47	Missense	p.T823P	10	31,812,927	A	C	ND
	SSA02	Frameshift	p.R988fs	10	31,815,779	-	GA	ND

ND, not determined

^*^NGS and/or Sanger conformation of somatic status in cases where normal tissue was available.

**Table 2:** Recurrent mutations in *JAK–STAT* and *PLCG1*.

**Gene**	**ID**	**Consequence**	**Amino-acid change**	**Chr**	**Position**	**Ref**	**Alt**	Somatic acquisition confirmed*
*JAK1*	SS87	Missense	p.Y654F	1	65,312,358	T	A	Yes
	SS29	Missense	p.L710V	1	65,310,560	G	C	Yes
*JAK3*	SS33	Missense	p.S989I	19	17,942,049	C	A	Yes
	SS47	Missense	p.Y1023H	19	17,941,341	A	G	Yes
*STAT3*	SS29	Missense	p.I498V	17	40,476,837	T	C	Yes
	SS37	Missense	p.Y640F	17	40,474,482	T	A	Yes
*STAT5B*	SS55	Missense	p.N642H	17	40,359,729	T	G	Yes
	SS86	Missense	p.N642H	17	40,359,729	T	G	Yes
*PLCG1*	SS26	Missense	p.S345F	20	39,792,584	C	T	Yes
	SS31	Missense	p.S345F	20	39,792,584	C	T	Yes
	SS84	Missense	p.S520F	20	39,794,139	C	T	Yes
	SSG01	Missense	p.R1158H	20	39,802,370	G	A	Yes
	SS86	Deletion	p.VYEEDM1161V	20	39,802,378	GTGTATGAGGAAGACA	G	Yes
	SS9	Missense	p.E1163K	20	39,802,384	G	A	Yes

^*^NGS and/or Sanger conformation of somatic status in cases where normal tissue was available.
