# Supplementary material for: Genomic analyses reveal recurrent mutations in epigenetic modifiers and the JAK–STAT pathway in Sézary syndrome
Source: Nat Commun. 2015 Sep 29;6:8470. doi: 10.1038/ncomms9470 (PMC4598843; doi:10.1038/ncomms9470)
Supplement: Supplementary Information — Supplementary Figures 1-12 [file ncomms9470-s1.pdf]

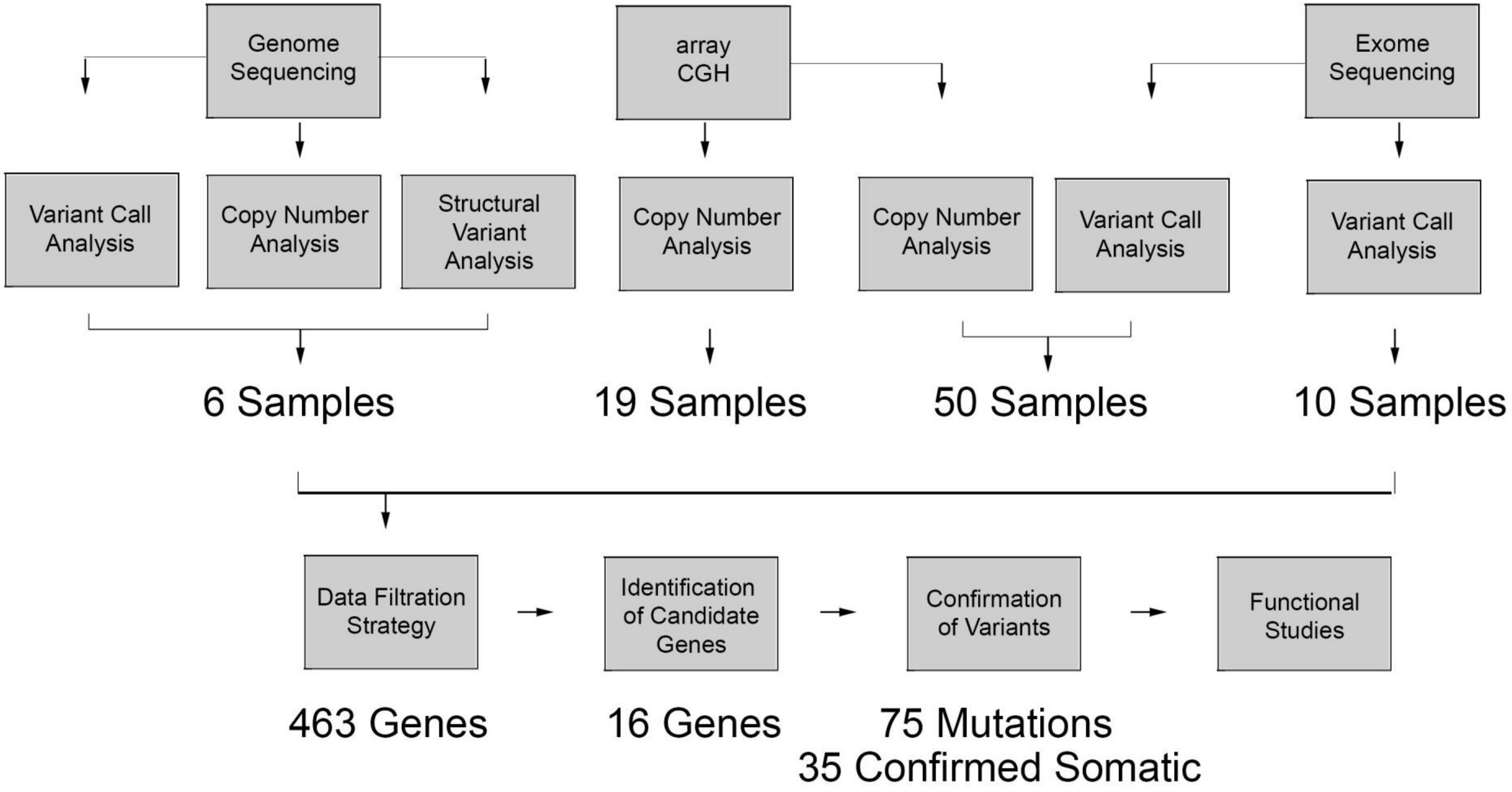

Supplementary Figure 1. Experimental paradigm. A combination of genome and exome sequencing coupled with array-comparative genome hybridization was performed on a total of 85 SS patients. Data filtration identified 463 genes with recurrent loss of function lesions. Of these, we focused our attention on 16 genes which included 75 mutations. For 35 of these mutations, we had matched normal tissue available to confirm the somatic nature of these mutations.

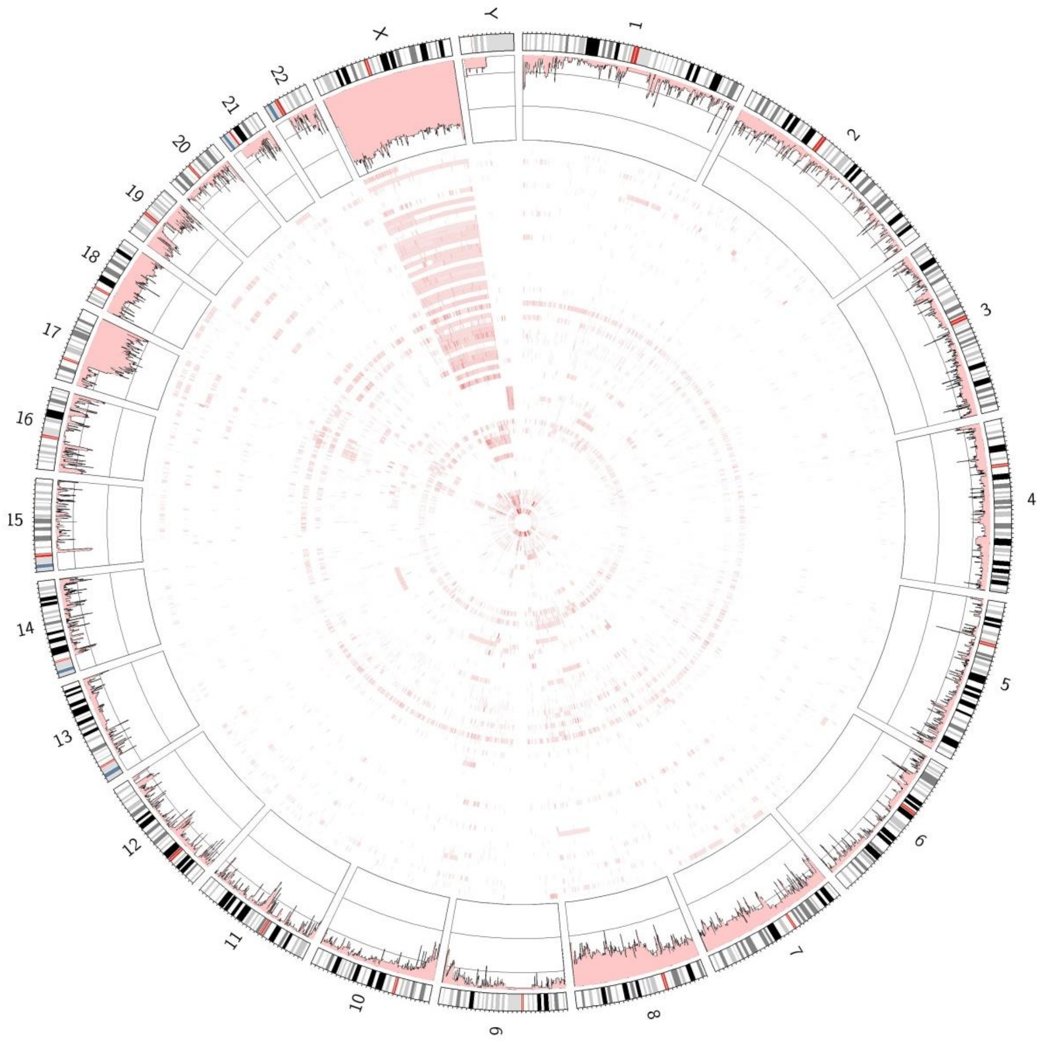

Supplementary Figure 2. Gains of chromosomal material identified in SS genomes by aCGH.

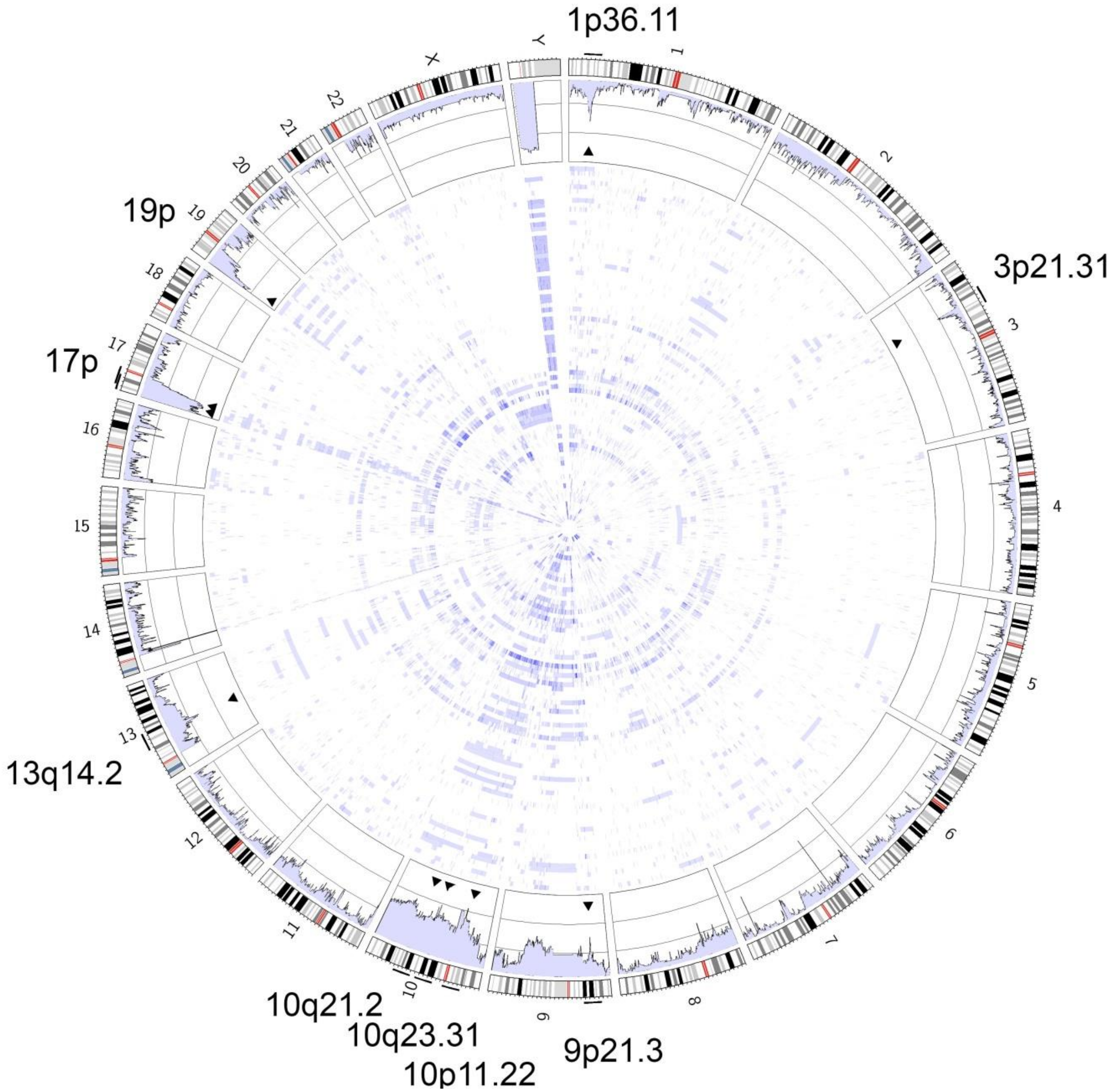

Supplementary Figure 3. Recurrent deletions identified by aCGH of SS genomes. Regions of interest (arrowheads) are highlighted by their chromosomal band co-ordinates.

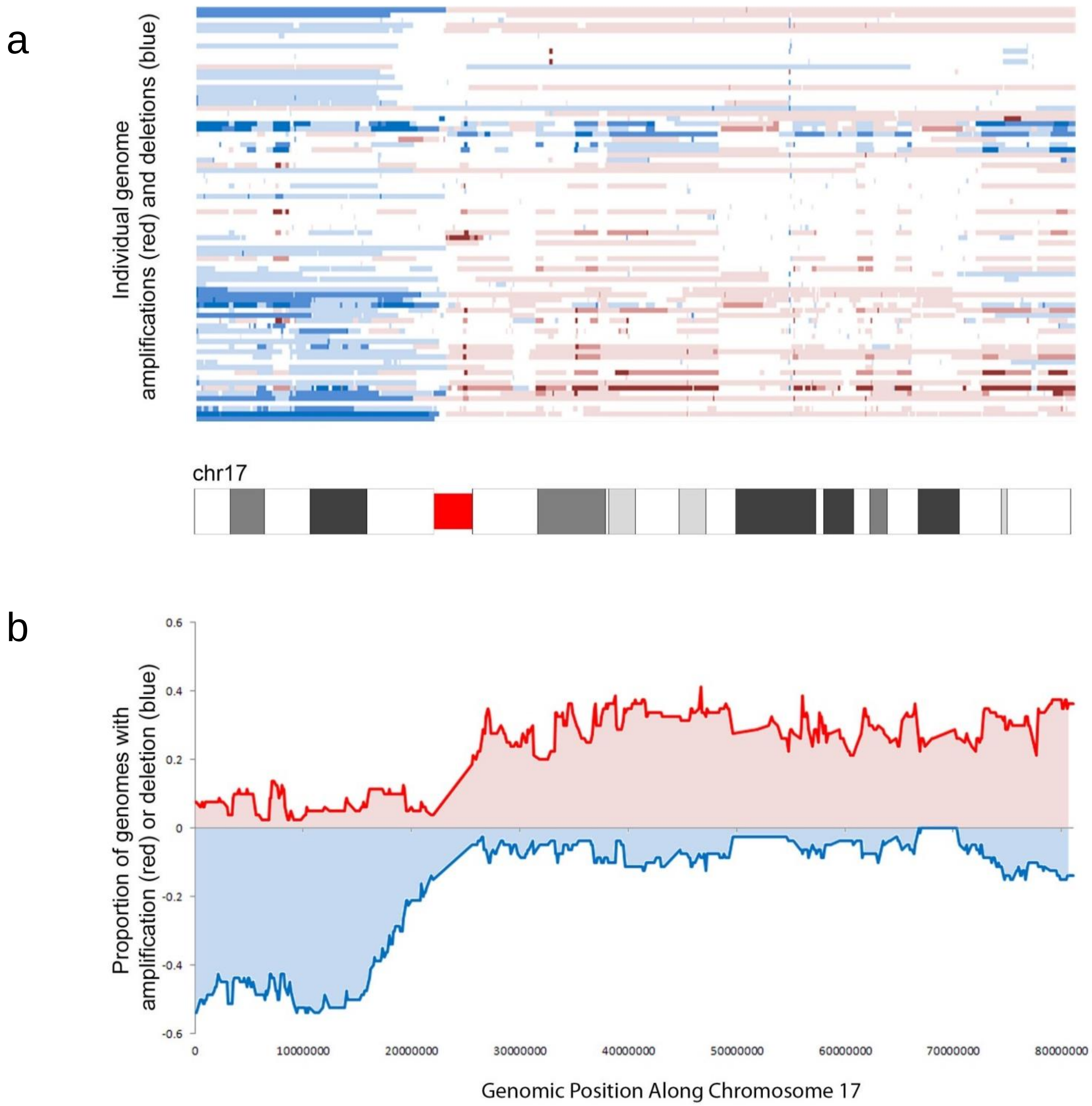

Supplementary Figure 4. Isochromosome 17 identified in majority of SS genomes. Data demonstrating loss of chromosomal material at the 17p locus overlying TP53 for individual SS genomes (a) and in aggregate for all SS genomes analyzed (b) are shown.

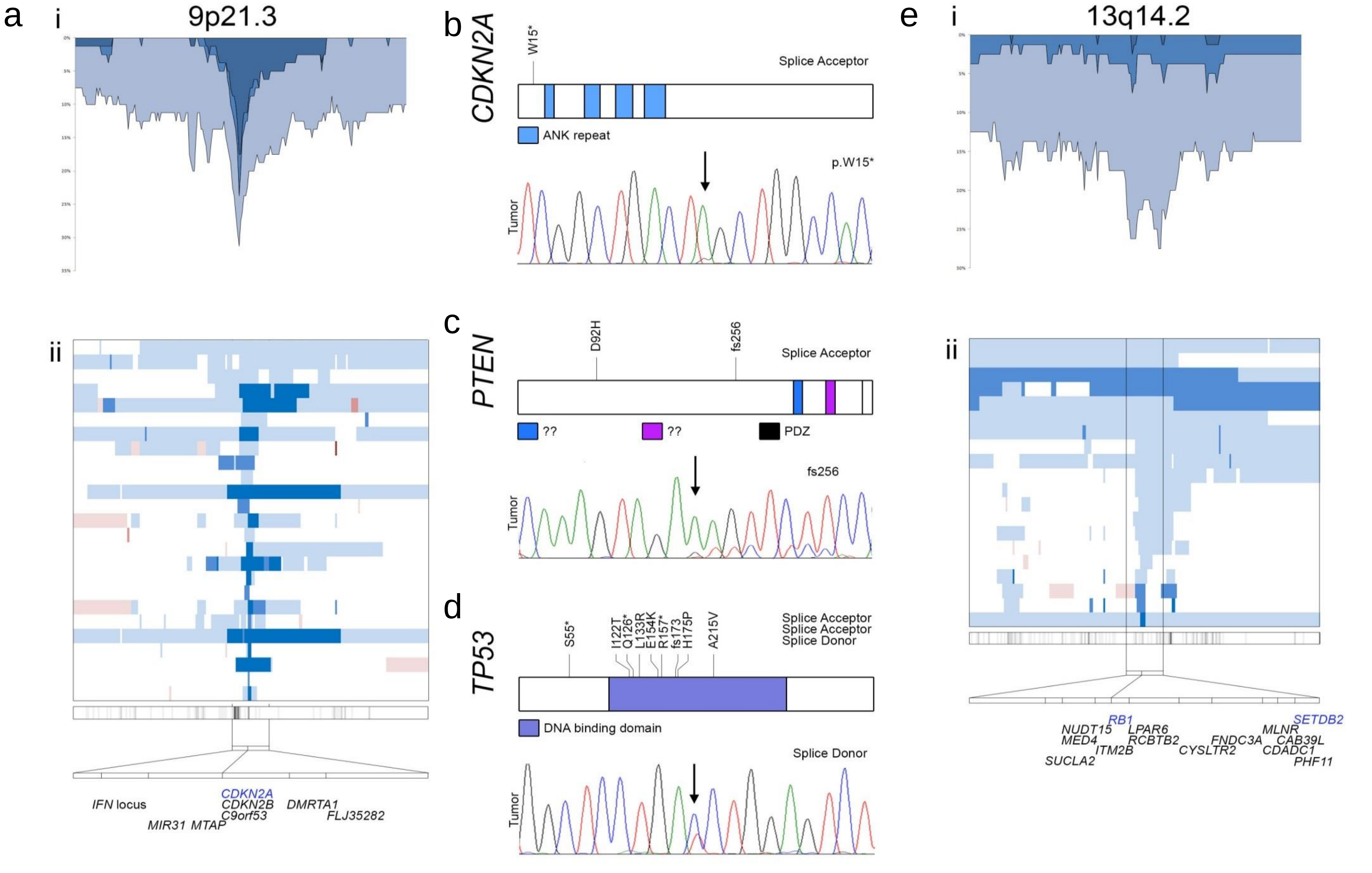

Supplementary Figure 5. Recurrent loss-of-function of tumor suppressor A summary of the total proportion of genomes (top, a and e) affected by loss of chromosomal material at the indicated gene locus is shown for CDKN2A and RB1 on chromosomes 9p21.3 and 13q14.2, respectively. Loss of chromosomal material for individual SS genomes is shown at bottom (a and e). A summary of the mutations identified in known tumor suppressor genes CDKN2A, PTEN and TP53 is shown (b-d) with functional protein domains highlighted for each.

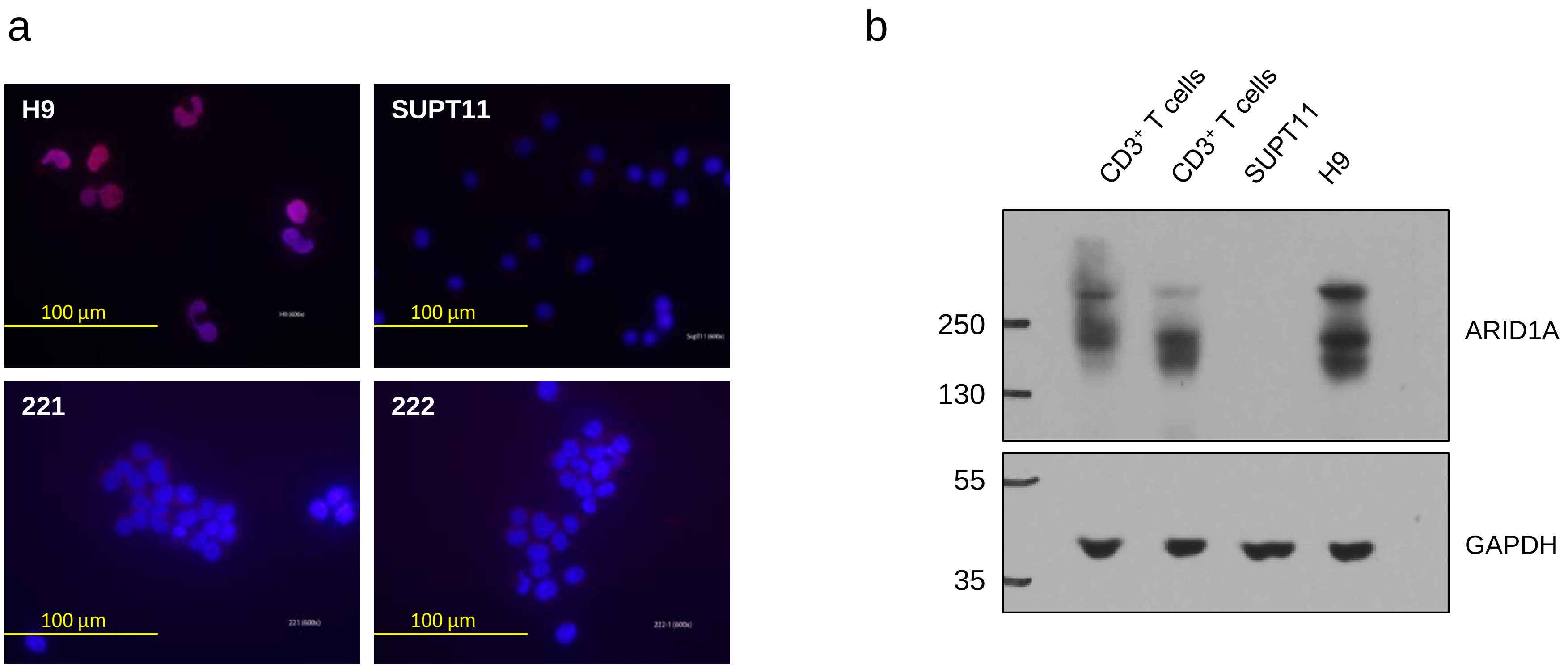

Supplementary Figure 6. Functional validation of loss of trithorax group components in SS. (a) Immunofluorescence staining of two clinical samples of SS exhibiting loss-of-expression of ARID1A. H9 cell line is used as positive control of expression of ARID1A while SUPT11 cell line is used as negative control. (b) Western blotting demonstrate that H9 cell line and human CD3+T cells express ARID1A while SUPT11 cells express very low level of ARID1A.

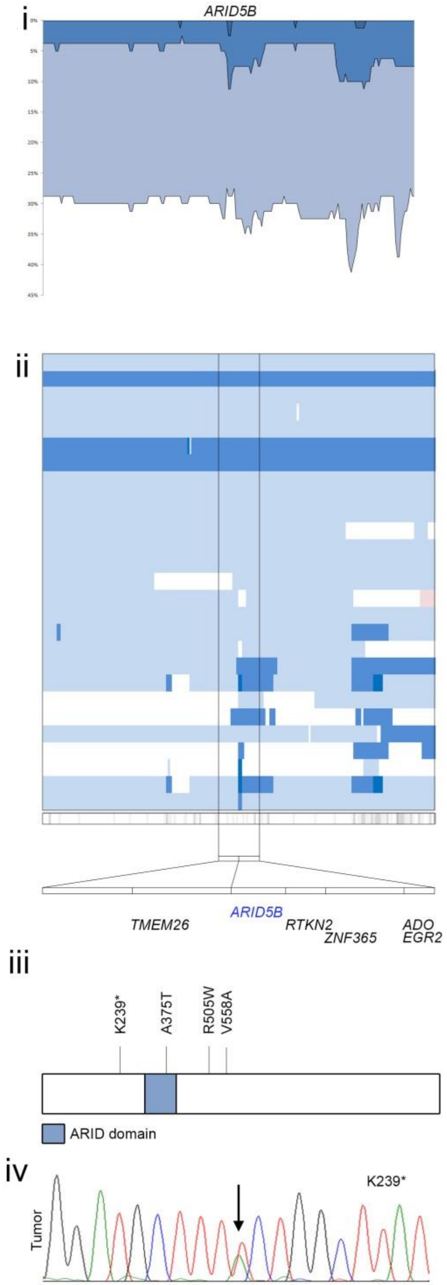

Supplementary Figure 7. Frequent loss-of-function of ARID5B by deletion and/or mutation. A summary of the proportion of all SS genomes showing loss of chromosomal material at this locus is shown (i, total; ii, individual). Mutations identified by whole exome or whole genome sequencing are shown (iii) and in many cases confirmed to be somatic (iv; Table 1).

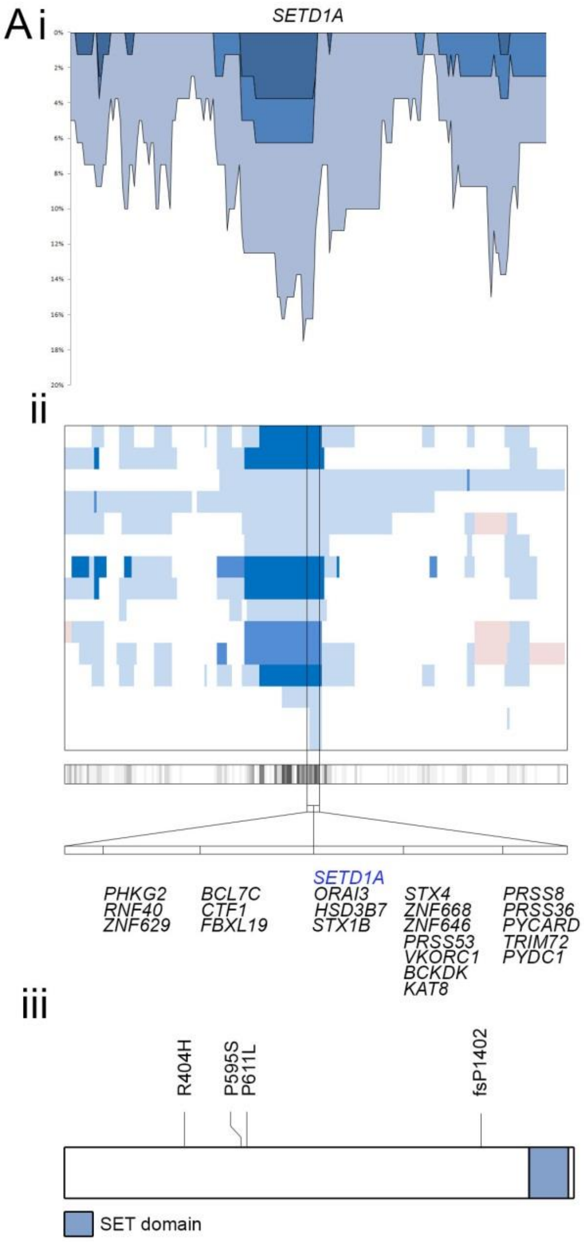

Supplementary Figure 8. Frequent loss-of-function of SETD1A by deletion and/or mutation. A summary of the proportion of all SS genomes showing loss of chromosomal material at this locus is shown (i, total; ii, individual). Mutations identified by whole exome or whole genome sequencing are shown (iii) and in many cases confirmed to be somatic (iv; Table 1).

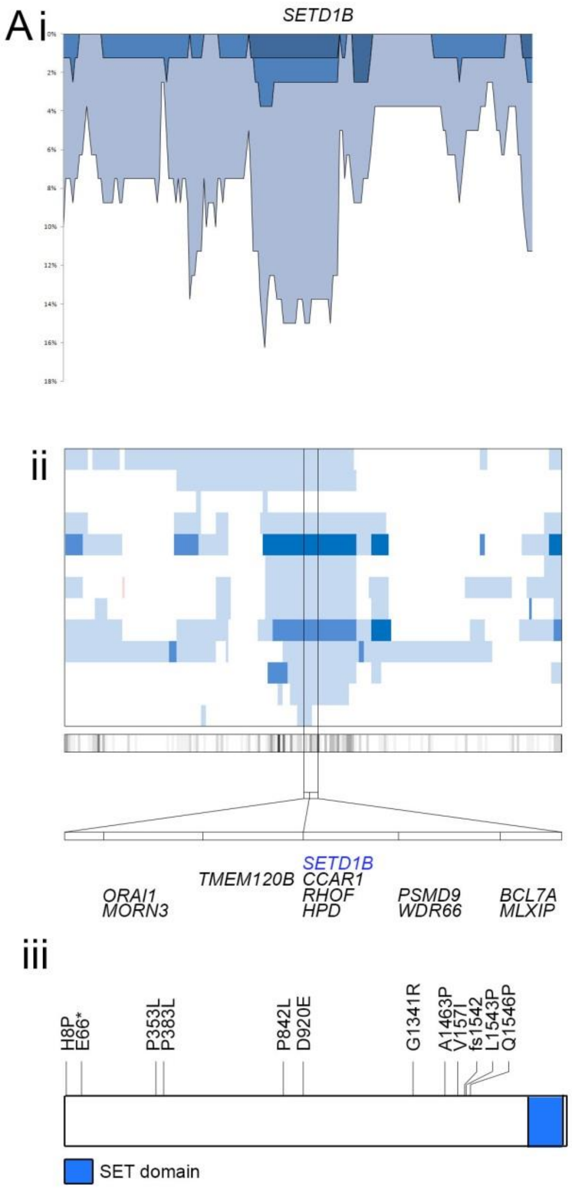

Supplementary Figure 9. Frequent loss-of-function of SETD1B by deletion and/or mutation. A summary of the proportion of all SS genomes showing loss of chromosomal material at this locus is shown (i, total; ii, individual). Mutations identified by whole exome or whole genome sequencing are shown (iii) and in many cases confirmed to be somatic (iv; Table 1).

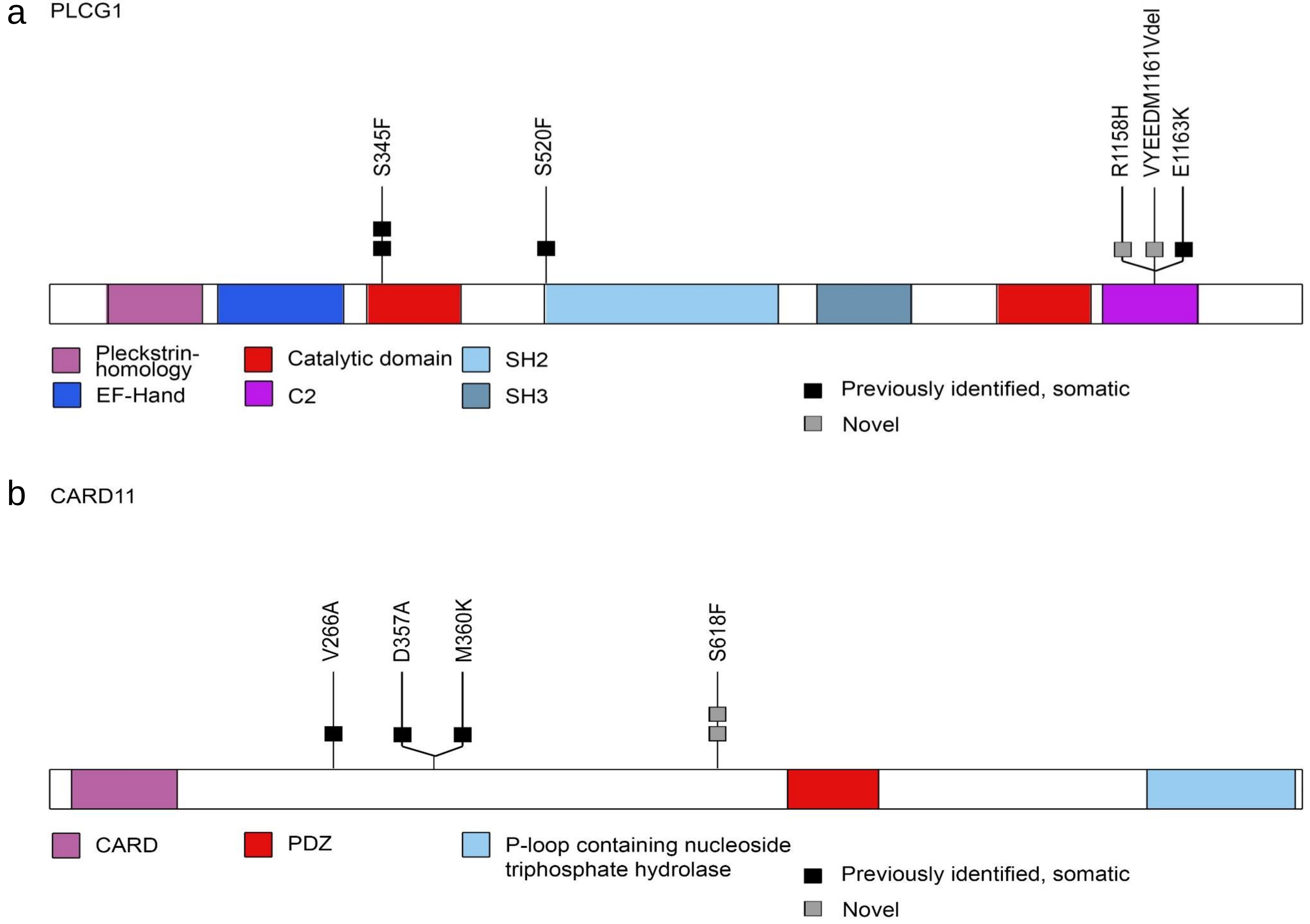

Supplementary Figure 10. PLCG1 and CARD11 mutations identified in primary SS samples. (a) Schematic representations of mutations in *PLCG1* identified in primary SS samples. (b) Schematic representation of mutations in *CARD11* identified in primary SS samples.

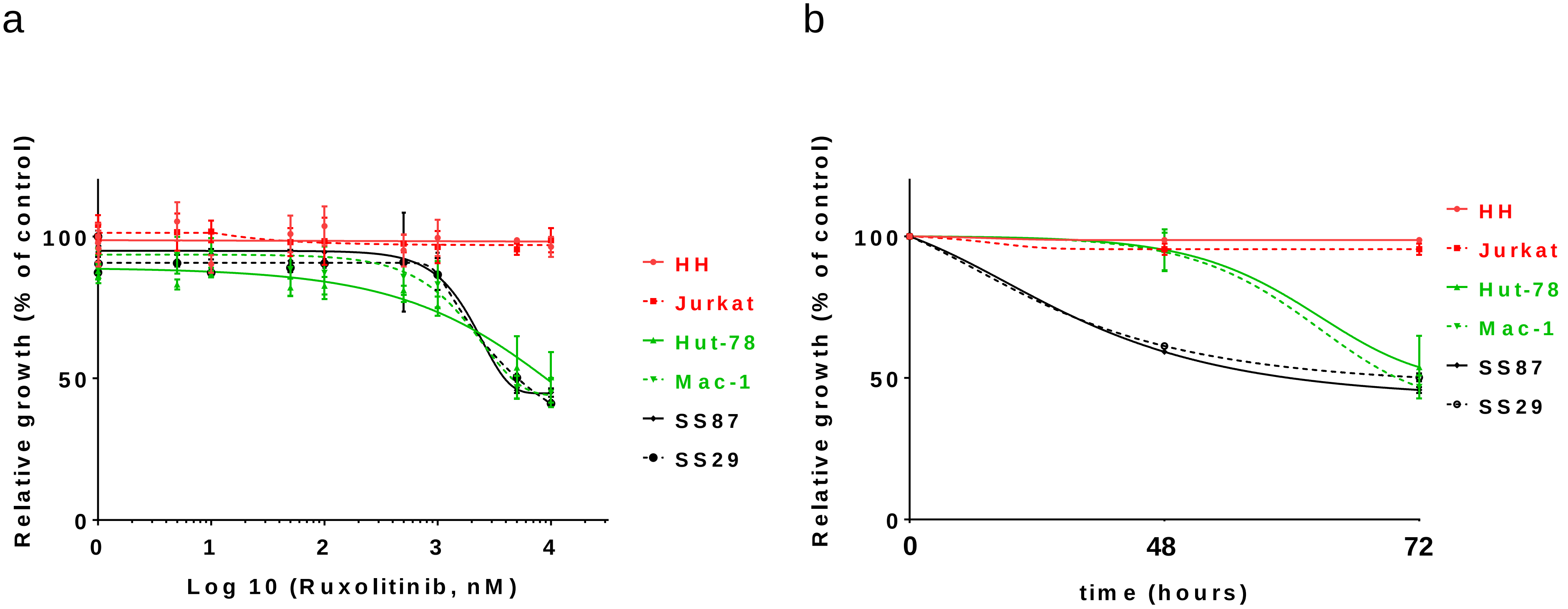

Supplementary Figure 11: Dose and time dependent responses to ruxolitinib treatment of Sézary syndrome (SS). (a) Dose-response curves for 2 primary SS leukemic cells (SS29 and SS87), the negative control cell lines (HH and Jurkat) and the positive control cell lines (Mac-1 and Hut-78) after 48 hours of treatment with ruxolitinib. (b) Time-response curve for the 2 primary Sézary syndrome leukemic cells (SS29 and SS87), the negative control cell lines (HH and Jurkat) and the positive control cell lines (Mac-1 and Hut-78) treated with 5µM of ruxolitinib.
All experiments were done in biological triplicates. The error bars represent the standard deviations for each measurement.

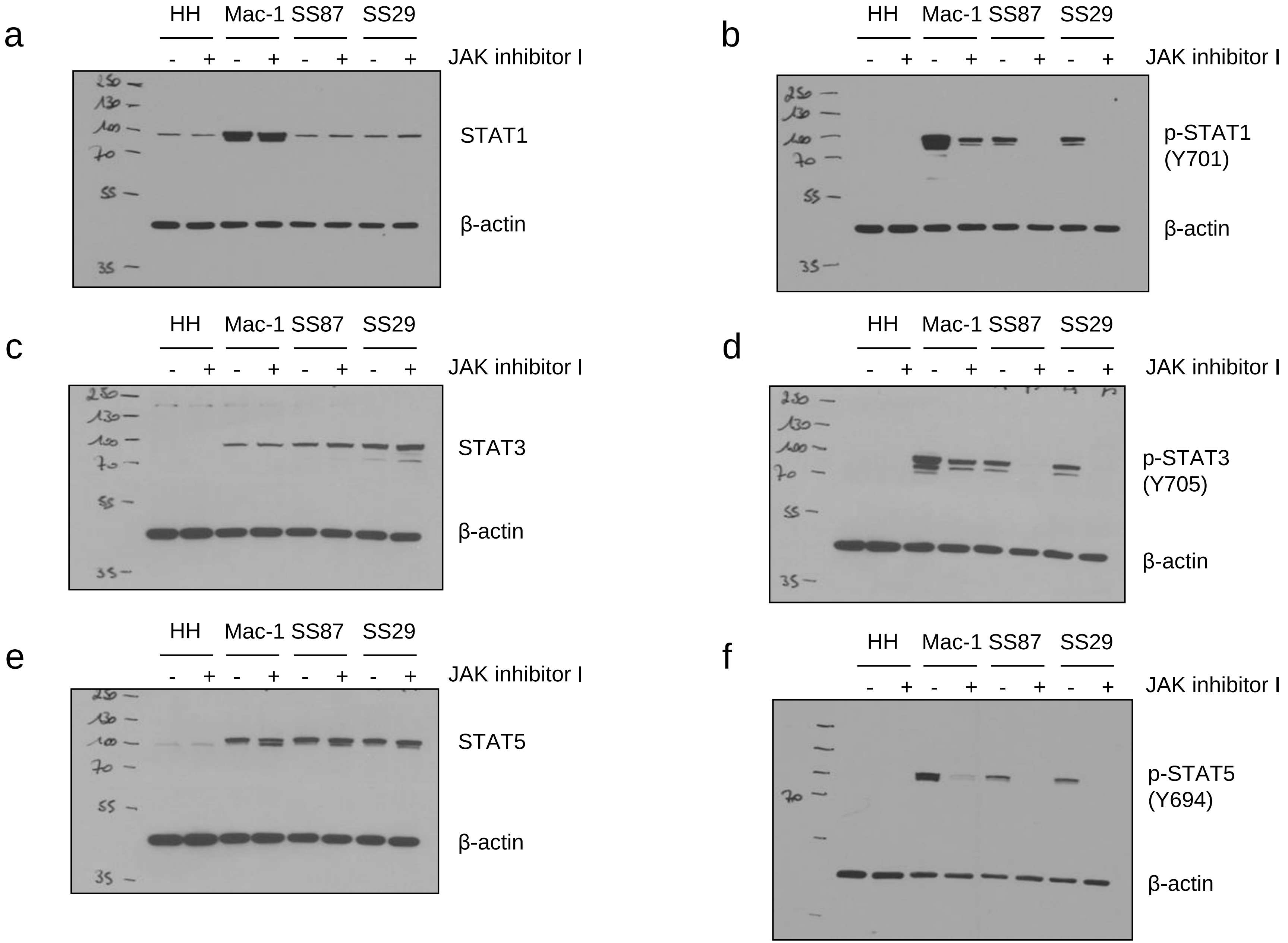

Supplementary Figure 12: Uncropped scans of western blot results displayed in Figure 4. (a) western blot results for STAT1. (b) Western blot results for pY701 STAT1. (c) Western blot results for STAT3. (d) Western blot results for pY705 STAT3. (e) Western blot results for STAT5. (f) Western blot results for pY694 STAT5.
